# seq-seq-pan: building a computational pan-genome data structure on whole genome alignment

**DOI:** 10.1186/s12864-017-4401-3

**Published:** 2018-01-15

**Authors:** Christine Jandrasits, Piotr W. Dabrowski, Stephan Fuchs, Bernhard Y. Renard

**Affiliations:** 1 Robert Koch Institute, Nordufer 20, Berlin, 13353 Germany; 2 Robert Koch Institute, Wernigerode Branch, Burgstraße 37, Wernigerode, 38855 Germany

**Keywords:** Pan-genome, Data structure, Whole genome alignment

## Abstract

**Background:**

The increasing application of next generation sequencing technologies has led to the availability of thousands of reference genomes, often providing multiple genomes for the same or closely related species. The current approach to represent a species or a population with a single reference sequence and a set of variations cannot represent their full diversity and introduces bias towards the chosen reference. There is a need for the representation of multiple sequences in a composite way that is compatible with existing data sources for annotation and suitable for established sequence analysis methods. At the same time, this representation needs to be easily accessible and extendable to account for the constant change of available genomes.

**Results:**

We introduce seq-seq-pan, a framework that provides methods for adding or removing new genomes from a set of aligned genomes and uses these to construct a whole genome alignment. Throughout the sequential workflow the alignment is optimized for generating a representative linear presentation of the aligned set of genomes, that enables its usage for annotation and in downstream analyses.

**Conclusions:**

By providing dynamic updates and optimized processing, our approach enables the usage of whole genome alignment in the field of pan-genomics. In addition, the sequential workflow can be used as a fast alternative to existing whole genome aligners for aligning closely related genomes.

seq-seq-pan is freely available at https://gitlab.com/rki_bioinformatics

**Electronic supplementary material:**

The online version of this article (doi:10.1186/s12864-017-4401-3) contains supplementary material, which is available to authorized users.

## Background

Thanks to the continuous advances in next generation sequencing (NGS) technologies the number of sequenced whole genomes is also continuously increasing. This has led to a 10,000 fold increase in available bacterial genomes over the past 20 years [1]. As a result complete sequence information for many species and phylogenetic clades has become available. The current approach to handle the diversity of sequences within a single population is to define a single reference genome with an accompanying comprehensive catalog of variants and other variable genome elements present within that population [2]. Unfortunately, this representation is limited, as complex genetic differences such as large deletions, insertions or rearrangements cannot easily be expressed in relation to a single reference genome [3]. This presents a significant drawback, since a combined representation of all genomic content of a species or population that captures the full information on similarity and variation between individual genomes is essential [4]. Therefore, the more versatile concept of using multiple instead of a single reference genome for common analyses of NGS data is attracting more and more attention.

Initially defined to be the sum of core and dispensable genes of all strains of one bacterial organism [5], the term pan-genome is now more commonly used to describe any set of associated sequences aiming for a collective analysis. Gathered under a newly evolving field termed computational pan-genomics, several methods for the generation of data structures that can represent a set of multiple sequences have been developed. These data structures generally aim to fulfill the following requirements: (i) easy construction and maintenance, (ii) adding and retrieving of (biological) information, (iii) comparison to other sets of genomes or short or long sequences from individuals, (iv) easy visualization and (v) advanced data storage [4].

We assessed a collection of tools applied for the analysis of multiple sequences (Table 1). Many of these tools use graphs to represent the pan-genome and focus on efficiently building and storing that graph [2, 6–9]. Some [10–12] focus on subsequent analyses such as mapping reads to the pan-genome, while others [13, 14] improve variant detection by using a set of reference sequences instead of a single one. The final category in our collection is made up by tools that introduce a complete data structure and provide methods for the construction, storage, processing and visualization of the pan-genome [3, 13, 15–17]. Most of these tools depend on information on the (dis-)similarity of genomes from a multiple genome alignment or a reference sequence with an adjoining corresponding set of variants to create a pan-genome. This prerequisite cannot represent structural variants (e.g. large deletions or insertions or rearrangements of sequences) in most cases and has to be obtained via external tools.

**Table 1:** Comparison of pan-genome tools. We analyzed tools for pan-genome analysis that are available or currently under development. This table lists the corresponding publications or websites. We compared the intended use cases of the tools and the prerequisite data required in order to use them. We evaluated the availability of features needed to work with the pan-genome in subsequent analyses, e.g. updating the set of included genomes. Furthermore, we assessed whether the proposed data structures take into account structural variants and whether it is possible to visualize the resulting pan-genome

Name	Objective	Input	Visualization	Structural	Functionality		
			of pan-genome	Variants	Update		Possibility to
					Add	Remove	include annotation
svaha [9]	Graph construction	Reference sequence + variants	External	Yes	No	No	No
cdbg [2]	Graph construction	Multiple reference sequences	External	Yes	No	No	No
cdbg_search [6]	Graph construction	Multiple reference sequences	External	Yes	No	No	No
SplitMEM [44]	Graph construction	Multiple reference sequences	External	Yes	No	No	No
TwoPaCo [7]	Graph construction	Multiple reference sequences	External	Yes	No	No	No
GCSA2 [8]	Graph indexing	Variation graph	No	No	No	No	No
GCSA [10]	Graph indexing	Reference sequence + variants	No	No	No	No	No
	Multiple sequence mapping						
BWBBLE [11]	Multiple sequence mapping	Reference sequence + variants	No	No	No	No	No
GenomeMapper [12]	Multiple sequence mapping	Reference sequence + variants	No	No	No	No	No
panVC [14]	Multiple sequence variant detection	Whole genome alignment	External	Yes	No	No	Yes
MHC-PRG [13]	Multiple sequence variant detection	Multiple sequence alignment	No	No	Yes	No	No
	Pan-genome data structure	AND variants					
GenomeRing [3]	Pan-genome data structure	Whole genome alignment	Yes	Yes	No	No	Yes
JST [15]	Pan-genome data structure	Reference sequence + variants	No	Yes	Yes	Yes	Yes
vg [17]	Pan-genome data structure	Reference sequence + variants	External	Yes	Yes*	Yes*	Yes
		OR multiple reference sequences					
PanCake [16]	Pan-genome data structure	Multiple reference sequences	External	Yes	Yes	No	No
		AND pairwise alignment					
seq-seq-pan	Pan-genome data structure	Multiple reference sequences	External	Yes	Yes	Yes	Yes

^∗^ Adding and removing of genomes in vg can be achieved using a combination of several steps

While four of the analyzed tools - JST [15], MHC-PRG [13], PanCake [16], and vg [17] - provide methods for adding or removing genomes from the pan-genome data structure, only GenomeRing [3], JST [15], panVC [14] and vg [17] offer the ability to annotate biological features. This is often caused by the representation of the pan-genome as graphs, for which there is no standard method providing a coordinate system, which severely complicates the use of existing annotation databases and formats. Proposed strategies for such coordinate systems [18] do not meet all preferential criteria (spatiality, readability, and backward compatibility) [4]. Additionally, new methods for essential analyses such as comparing genetic information of individual samples with a graph of reference sequences have to be developed.

Another (well-established) representation of sets of genomes is their alignment. Whole genome alignments (WGA) implicitly provide a coordinate system that allows the translation between pan-genome and strain genome position, enabling annotation of the alignment with biological features of the individual genomes. Due to the extensive research on whole genome alignment [19–28], standard formats (eXtended Multi-FastA [29] and Multiple Alignment Format [30]), and methods for processing and visualizing WGA results are available [3, 31–35]. In the field of pan-genomics, whole genome aligners are used for the analysis of a set of closely related non-collinear genomes (e.g. several strains of a bacterial species). These genomes contain large insertions and deletions and also rearrangements and inversions of sequences that have to be detected and aligned properly [19, 23]. Several methods (Mugsy [23], progressiveCactus [19], progressiveMauve [25] and TBA [27]) have been developed to meet this challenge.

In summary, WGA structures presently fulfill most of the desirable properties of a pan-genome, but a severe drawback of existing methods is their final, non-updatable alignment result.

We here present seq-seq-pan, a framework that enables the usage of WGA as a pan-genome data structure. We provide methods for adding additional genomes or removing them from a set of aligned sequences and use them to sequentially align whole genome sequences. Throughout the sequential process we take measures to optimize the resulting whole genome alignment and provide a linear representation that can be used in place of a reference genome with established methods for subsequent analyses such as read mapping and variant detection.

## Methods

### seq-seq-pan workflow

#### Overview

**Fig. 1:**
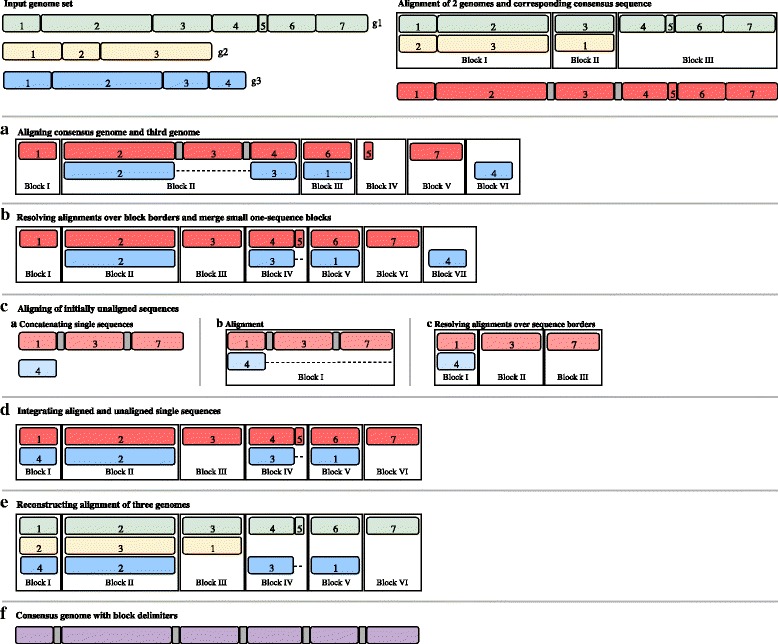
Visualization of the alignment workflow for an example with three genomes. Input genomes (g1-3) are depicted as green, yellow and blue blocks. All sub-sequences are part of locally collinear blocks (LCBs) in the final result and are therefore marked within the whole genomes and numbered according to their appearance in the respective genome. The first two genomes are aligned and provided as separated blocks of aligned sub-sequences. Block I and II indicate a rearrangement of sub-sequence 3 of g1 when compared to g2 and parts of g1 are not present in g2. Consensus sequences are built individually for each LCB in the alignment and concatenated with stretches of ’N’ as delimiters to form a consensus genome (depicted in red with delimiters in gray). It is used in the alignment with g3, which is presented in detail in steps a-e. **a** The consensus genome is aligned with the third genome (g3, blue), yielding six blocks. Block I and III represent a rearrangement of sub-sequence 6 of g1. Block II shows a large deletion in g3 compared to the consensus genome. Block IV-VI show single-sequence blocks. **b** Blocks resulting from alignment with the consensus genome are broken up into smaller blocks at delimiter positions (Block II in a is now Block II-VI in b). The small single-sequence block with sub-sequence 5 of the consensus genome (Block IV in a) is merged to its neighboring sub-sequence 4 of the consensus genome, introducing gaps into sub-sequence 3 of g3 (see Block IV in b). **c** Remaining single-sequence blocks of both genomes (depicted in lighter red and blue) are concatenated with stretches of ’N’ as delimiters (c.a). Sequences are aligned (c.b) and resulting blocks are resolved at delimiter positions (c.c). Small single-sequences would also be merged to neighboring blocks (not shown). **d** Aligned and single-sequence blocks from step c are joined with initially aligned blocks and all blocks are sorted by their position in the consensus genome. **e** The full alignment is traced back using the newly formed blocks and the alignment of the first two genomes. **f** A consensus genome is built from the full alignment and alignment of additional genomes is achieved by consecutive repetition of steps a-f

The key notion of seq-seq-pan is to use and optimize fast, well established whole genome alignment methods to construct a pan-genome from an a priori indefinite set of genomes. For this part, we use progressiveMauve [25], a fast whole genome aligner that accurately detects large genome rearrangements. The alignment result is comprised of a set of blocks of aligned sequences that are internally free from genome rearrangements (referred to as locally collinear blocks (LCBs)). For each LCB we derive a consensus sequence using the concept of majority vote and combine all sequences with delimiter sequences of long stretches of the character ’N’. These delimiters are inserted to prevent alignment of sequences over block borders in the following step, because blocks are not consecutive in all genomes. After alignment of the consensus genome with the subsequent genome in the set, all LCBs stretching over block borders are separated. Unaligned sequences of each genome are analyzed again, to align sequences that are considered to be contextually unrelated [25]. The resulting LCBs with sequences from one or both genomes are joined to the previously aligned blocks. The complete alignment of all genomes is reconstructed from the current and prevenient alignment. Optimizing measures are taken throughout the workflow to maintain the synteny of the original genomes and avoid accumulation of short, unrelated sequence blocks (Fig. 1). Repetitive sequences within genomes are not aligned with each other but integrated into the alignment and its linear representation as they appear in the original genomes.

Below we describe all steps of the whole workflow in detail. The details of implementation and consecutive order of individual steps are depicted in Additional file 1.

#### Consensus genome construction

LCBs are combined into a consensus genome by concatenating the consensus sequence of each block. At each position within the LCB, all aligned sequences are compared and the most abundant base is chosen for the consensus sequence. In case of ties, the base is drawn randomly from the available choices. To prevent alignments across block borders when the consensus genome is used for alignment, we integrate a sequence of 1000 ’N’ (undefined nucleic acid) between the consensus sequence blocks into the final sequence. In addition to the consensus genome, two accompanying index files are created. One contains the start positions of all delimiter sequences within the consensus genome and therefore enables the reconstruction of the alignment of all genomes from the alignment of the consensus genome with an additional sequence (referred to as “consensus index file”). The second index file contains the coordinates of all gaps of all sequences per block in the consensus genome, improving the performance of the reconstruction step and the mapping of coordinates between genomes. Furthermore, we make note of the sequence identifier and the description of all genomes and chromosomes, as this information is not contained in the final output of progressiveMauve.

#### Alignment step

When aligning two sequences with progressiveMauve [25], the alignment is partitioned in locally collinear blocks to allow for the representation of structural differences such as inversions or translocations. Each resulting block contains parts of either both or one of the genomes locally collinear. They form the basis for the subsequent workflow steps. We chose progressiveMauve for this step as we introduce artificial insertions and rearrangements by representing the genomes as a linear consensus genome, which are accurately resolved by this whole genome aligner.

#### Merging step

Sequences specific for one of the genomes are also reported as LCBs, but these only contain parts of this one genome. LCBs containing only a single unaligned sequence are typically moved to the end of the alignment file. They are created to ensure collinearity within blocks and can sometimes be of small length. When used in a sequential workflow, it can be advisable to avoid assembling short one-sequence-LCBs and attaching all of them to the end of the consensus genome. We prevent the accumulation of small blocks by merging short one-sequence-LCBs with their neighboring blocks within the genome and realigning consecutive gap stretches (see “Realignment step” section). These short blocks can not only emerge in the alignment step but also result from the resolving step, when a LCB is split at block border positions (see “Resolving step” section).

#### Realignment step

Alignment is improved by realigning genomes at sites where a gap ends in one sequence and starts in the other (referred to as “consecutive gaps”). We scan through the whole alignment and identify all positions with consecutive gaps. Then we extend the interval to the sequence on both sides of the gap sequences by the length of the longer sequence or up to block borders and align these sequences again.

#### Resolving step

Aligning a genome with a consensus genome can result in alignments that span the borders of the LCBs making up the consensus genome. We identify these blocks using the consensus index file. Then, we split them at the start and end of the delimiter sequence. If the alignment spans a complete delimiter sequence the separation results in three new blocks: the first and third one contain the aligned sequences of the two genomes. The second one includes only the sequence of the new genome that was aligned to the delimiter sequence. All gaps contained in this block are removed. In cases where the delimiter sequence is matched with a gap sequence only, we discard the complete block.

#### Alignment of initially unaligned sequences

We take the forward representation of all one-sequence-blocks per genome and sort them. We concatenate the sequences, again integrating stretches of 1000 ’N’, ending up with one sequence for each of the genomes. These sequences are then aligned using the same process as with the full genomes. Alignment with progressiveMauve, the optional Merging Step and the Realignment Step, are followed by a two-step Resolving and Reconstruction process using each of the initially concatenated sequences as “consensus sequence” (see Fig. 1 and Additional file 1). All blocks with newly aligned and unaligned sequences are joined with the initially aligned blocks for the final Reconstruction step.

#### Reconstruction step

All previous steps result in a set of LCBs that contain parts of the consensus genome, the aligned genome or both. For all LCBs that include consensus genome sequences, we reconstruct the alignment that formed this consensus sequence in the previous workflow iteration. For this we use the coordinate system of the consensus genome and the index file containing delimiter sequence positions. We translate the start and end positions of the consensus sequence in each LCB to their positions within the original genomes. With this information we can extract the bases, gaps and positional information of all sequences and report the complete alignment of all genomes for the current workflow iteration.

#### Removing a genome

After removing a genome from a pan-genome, gaps that were introduced only for the alignment of the removed genome are cut from the remaining genomes. Adjacent LCBs that are now composed of consecutive regions of the same set of genomes are joined to form one LCB.

### Setup for comparison experiments

#### Data

We use several sets of reference genomes available in the NCBI RefSeq database as of November 30^*th*^, 2016 for our experiments. The set of 43 *Mycobacterium tuberculosis* genomes is used throughout all experiments. To demonstrate the ability of seq-seq-pan to align a large number of genomes, we use the set of all *Staphylococcus aureus* and *Escherichia coli* reference genomes. These sets contain 144 and 207 genomes, respectively. Accuracy of alignment was tested on a simulated dataset of twelve genomes with the genome of *E. coli* K12 as basis for the simulation of evolution. For evaluating the run-time when adding an additional genome to a pan-genome, we used another *M. tuberculosis* genome that became available on December 26^*th*^, 2016 (for details on genomes see Additional file 2).

#### Simulated data

**Fig. 2:**
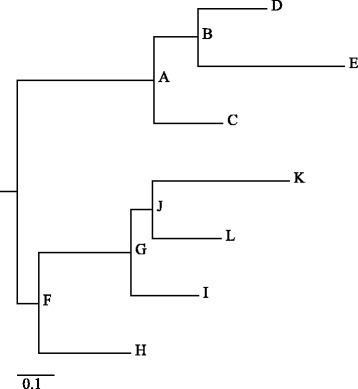
Visualization of the phylogenetic tree used to simulate genomes with EVOLVER. The corresponding NEWICK tree is (((D:0.015625,E:0.0333)B:0.01,C:0.015625)A:0.03125, (((K:0.03125,L:0.015625)J:0.005,I:0.015625)G:0.02083, H:0.02083)F:0.005);. (drawn with online version of Phylodendron [43])

Accuracy of alignment was tested on a simulated dataset of 13 genomes. For the simulation of genomes with a known true alignment we used the EVOLVER software [36] and the evolverSimControl suite [37] as described in the Alignathon project [38]. The tool evolverSimControl enables the user to simulate several genomes along a phylogeny with EVOLVER. We used an *E. coli* K12 genome (NC_000913.3) as the origin of the evolution simulation. For the evolution parameters we adapted the example provided by the EVOLVER team. We fit the parameters to the smaller size of the *E. coli* genome by changing the probabilities of large insertion and deletion events and setting the maximum size of these events to 7000 – roughly the size of the longest *E. coli* gene. We simulated twelve genomes without using mutation acceptance constraints with the phylogeny depicted in Fig. 2.

#### Comparison of alignments

We use an alignment comparison method to compare our results with the results of other whole-genome aligners: the tool mafComparator from the mafTools collection [38]. To compare the alignments of the simulated dataset with the true alignment, we calculate recall, precision and F-score as described in the Alignathon project [38]. To use the same method for comparing alignments of the *M. tuberculosis* we choose the alignment of the other aligners to act as the true alignment and our results as the prediction in each comparison. Here, we use the F-score to assess the similarity of the alignments. As in this case there is no ground truth to compare our results to we derive the accuracy of our alignment method from comparison to four other alignment tools.

#### Sorting and merging

Due to the sequential nature of our workflow, the order in which genomes are added to the pan-genome might influence the resulting alignment. When additional genomes are added to existing alignments, they can not be set in relation to the genomes that are part of the alignment, but have to be added “on top” of them. Therefore, the alignment process should yield similar alignments irrespective of the order of genomes. To investigate the effect of sorting we arrange the genomes by similarity and by consecutive dissimilarity and compare the results. To sort the input genome sequences by similarity we apply the D2z score [39] on all pairs of sequences. The score reflects the sequence similarity, i.e. higher scores stand for more similar sequences. We calculate the upper quartile of all similarity scores and select the genome with the smallest distance from all others. The remaining sequences are ordered by their similarity to this genome.

As an alternative, to obtain a series of strongly differing genomes, we sort them as follows: we again start with the genome with the smallest distance from all others. Then we choose the one with the lowest similarity score as second and the genome most similar to the first for the third position and continue to alternate genomes in this manner throughout the complete set (so the sequence 1, 2, 3, 4, 5, 6 becomes 1, 6, 2, 5, 3, 4).

Additionally we constructed the alignment of the simulated dataset and the *M.tuberculosis* dataset a hundred times and the larger datasets (*S. aureus* and *E. coli*) 10 times with randomly sorted genomes and compare them to the alignment using genomes sorted by similarity. We also compare alignments that were created with and without using the merging steps (See order of genome sets in Additional file 2).

#### Whole genome alignment tools

We compared our sequential genome alignment approach with whole genome alignment tools to review the accuracy of the final alignment. For this, we chose progressiveMauve [25], Mugsy [23], progressiveCactus [19] and TBA [27] as these are commonly used methods for WGA that allow aligning non-collinear genomes with large deletions and insertions, inversions and rearrangements. Each of these tools separates the final alignment into LCBs. We parametrized all tools to not report duplications and disabled filters on LCB sizes to fit the results to the methology of seq-seq-pan. In addition to comparing the final alignments, we analyze the time and memory needed to create these results. In cases where no ground truth for the alignment is available, we regard the concordance of the results of all tools.

#### Pan-genome tools

For comparison, we choose PanCake, which also accepts whole genomes as input and bases the construction of the pan-genome data structure on sequence alignment methods. PanCake represents all genomic sequences in the form of feature instances. Each feature contains part of a genome sequence and start and stop coordinates within the genome. By using the information of pairwise genome alignments, shared features can be extracted. These features contain a single version of the sequence and a list of edit operations and positional information describing all aligned sequences. Following the recommendations by the authors, nucmer [24] was used for pairwise alignments. We measure the time it takes to construct a pan-genome. Tasks that are part of many analyses of pan-genomes include adding an additional genome or removing a genome and extracting a genomic sequence from the pan-genome. Thus, we examine whether these steps are possible and which run-time they require. To account for differences in time needed to extract genomes based on their position within the pan-genome, we extracted each genome once and calculated the average time.

## Results

As we sequentially construct a whole genome alignment, we compare our results with the alignments of progressiveMauve [25], Mugsy [23], progressiveCactus [19] and TBA [27] for all datasets. We compare the run-time and memory requirements of pan-genome construction and the provided functional features between seq-seq-pan and PanCake [16] using the *M. tuberculosis* dataset. We show that the order of genomes has minimal effects on the final alignment and that the merging step produces a less fragmented alignment. For all comparison analyses, we show the results for the whole genome alignment constructed with seq-seq-pan from genomes sorted by similarity using the merging step.

### Sorting and merging

**Fig. 3:**
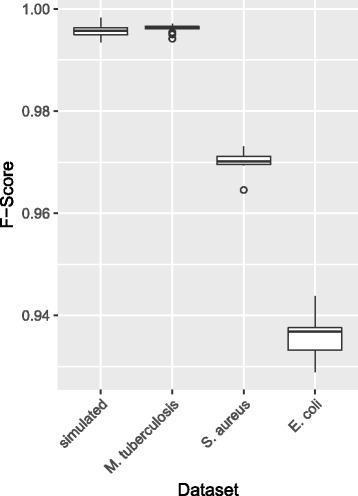
F-scores for comparing alignments using different sort orders for genomes. Genomes of each dataset were sorted by similarity and dissimilarity and randomly (100 times for the simulated and *M. tuberculosis* datasets and 10 times for the *S. aureus* and *E. coli* datasets) and aligned using the sequential workflow. The F-score is used as measure of consistency for alignment when comparing alignments with the dissimilar and random sort orders to the alignment with genomes sorted by similarity. All F-scores were similar within datasets and greater than 0.93 for all comparisons

We use 102 different orders for the simulated and the *M. tuberculosis* dataset and 12 different orders for the larger *S. aureus* and *E. coli* datasets and compare the results for all sort orders with the alignment using the genomes sorted by similarity. For these comparisons we use the mafComparator tool from the mafTools suite [38] and use the F-score for the assessment of the alignments similarity. As shown in Fig. 3 the order of genomes has minimal effect on the resulting alignment.

For investigation of the effects of the merging steps we use the simulated and the *M. tuberculosis* dataset with genomes sorted by similarity. Using the sequential workflow without the merging step, results in the alignment of fewer genomes within each LCB and a high number of single-sequence blocks in both datasets (Table 2). This indicates that the alignment is more fragmented when small LCBs are not merged to their neighboring blocks. Nevertheless, the F-score comparing the results with and without merging with the truth in the simulated dataset indicates only small differences in the overall alignment (Table 2).

**Table 2:** Effect of merging short one-sequence LCBs

	Total alignment	Mean number of	Number of	Number of short	Precision	Recall	F-Score
	length	sequences in LCB	short LCBs	one-sequence LCBs			
Simulated dataset (13 genomes)							
With merging step	4809015	9.2	0	0	0.993	0.475	0.643
Without merging step	4789770	5.5	318	156	0.993	0.475	0.643
*M. tuberculosis* dataset (43 genomes)							
With merging step	4826979	16.1	0	0	-	-	-
Without merging step	4859842	7.5	154	109	-	-	-

We compare the results from sequentially aligning two genome datasets including and excluding the merging step in the workflow. For estimation of the fragmentation of the alignment we compare the total alignment length, the number of sequences per block and the number of small (< 10 bp) LCBs and focus on the ones containing only sequences from one genome. By comparing the precision, recall and F-score of both alignments compared to the true alignment of the simulated dataset we show that the accuracy of the alignment is not affected by the merging step

### Comparison with whole genome alignment tools

The results of all whole genome aligners and our approach are competitive. In the simulated setting seq-seq-pan achieves similar precision and recall as progressiveMauve and Mugsy and better results than progressiveCactus (Table 3). We assessed whether the results of progressiveCactus and Mugsy improved when parametrized to detect duplications. This had almost no effect for Mugsy and improved the comparatively low precision for progressiveCactus, but reduced the recall. Our workflow achieves a precision as high as TBA, but all aligners show a significantly lower recall than TBA. However, comparably low recall values were also observed for simulated datasets used in the publication introducing the comparison method applied here [38]. For the *M. tuberculosis* dataset, the result of seq-seq-pan is most similar to the alignment by Mugsy and closer to the one from TBA, the most accurate aligner for the simulated dataset, than all other tested aligners. ProgressiveCactus shows the least concordance with all aligners, but all F-scores for comparison between all aligners are greater than 0.9 (Table 4).

**Table 3:** Precision, Recall and F-Score for alignments of the simulated dataset

	Precision	Recall	F-score
TBA	0.993	0.999	0.997
progressiveMauve	0.992	0.477	0.644
seq-seq-pan	0.993	0.475	0.643
Mugsy	0.999	0.474	0.643
Mugsy with duplications	0.999	0.474	0.643
progressiveCactus	0.892	0.473	0.618
progressiveCactus with duplications	0.999	0.339	0.506

We compare the results of all alignment tools with the true alignment of the simulated genomes. Aligners are sorted first by F-score and then by Recall

**Table 4:** F-score for pairwise comparison of alignment results for the *M. tuberculosis* dataset

	seq-seq-pan	Mugsy*	progressiveMauve	TBA	progressiveCactus
seq-seq-pan	-	**0.996**	**0.991**	**0.975**	**0.934**
Mugsy*	**0.996**	-	0.990	0.974	**0.934**
progressiveMauve	**0.991**	0.990	-	0.972	0.928
TBA	**0.975**	0.974	0.972	-	0.914
progressiveCactus	**0.934**	**0.934**	0.928	0.914	-

We estimate the similarity of alignments of progressiveMauve, Mugsy, progressiveCactus, seq-seq-pan and TBA, by calculating the pairwise F-score. The aligner with the most similar alignment is shown in bold for each aligner. * Aligning 43 *M. tuberculosis* genomes caused a segmentation fault in Mugsy. We were able to align 39 genomes and therefore compare the results only for this set of sequences

Table 5 shows the high speed up seq-seq-pan achieves compared to the whole genome alignment tools. Seq-seq-pan aligns 13 simulated genomes within 30 min and 43 *M. tuberculosis* genomes within two hours - being at least five times faster than all other tools with the real data set. ProgressiveCactus required almost two days for the alignment of 43 genomes and we were unable to align the whole set with Mugsy. It took Mugsy almost 15 h to align 39 (randomly chosen) genomes. TBA requires pairwise alignments for all genomes in the dataset and builds the alignment on top of these. Table 6 shows the cumulative run time for all steps in the alignment workflow. For alignment of the simulated dataset ${\binom {13}{2}=78}$ pairwise alignments with a mean run time of 4 min 29 s were calculated, and for the *M. tuberculosis* dataset, ${\binom {43}{2}=903}$ pairwise alignments with a mean run time of 10 h 15 min were required. Of course, depending on the resources available, sets of pairwise analyses can be done in parallel.

**Table 5:** Run-time and memory usage. We compare seq-seq-pan to other whole genome aligners in terms of run-time and memory usage. Time and memory are indicated for single-threaded processes. Individual steps for TBA can be run in parallel

	Elapsed wall clock time (hh:mm)	Maximum resident set size (GB)
Simulated dataset (13 genomes)		
seq-seq-pan	00:30	0.77
progressiveMauve	02:33	4.93
Mugsy	01:08	1.01
progressiveCactus	03:41	1.00
TBA	04:59	0.34
*M. tuberculosis* dataset (43 genomes)		
seq-seq-pan	02:06	1.20
progressiveMauve	09:03	2.79
Mugsy*	14:52	3.26
progressiveCactus	47:09	5.54
TBA	386 days	1.32
*S. aureus* dataset (144 genomes)		
seq-seq-pan	08:55	4.27
*E. coli* dataset (207 genomes)		
seq-seq-pan	68:19	8.5

For the larger datasets (*S.aureus* and *E.coli*) only seq-seq-pan was used for the alignment due to run-time limitation of other tools. * Aligning 43 *M. tuberculosis* genomes caused a segmentation fault in Mugsy. This table lists data for aligning 39 genomes with Mugsy, but the whole set of 43 genomes for all other tools

**Table 6:** Comparison of seq-seq-pan and PanCake

	seq-seq-pan	PanCake	Nucmer
Time for construction (hh:mm:ss)	02:06:00	88:10:00	03:04:00
Maximum memory usage	1.20 GB	2.34 GB	0.10 GB
Pan-genome file size	198 MB	36 MB	-
Time to add genome	00:04:01	05:33:52	00:08:48
Mean time for extraction of sequence*	00:00:09	00:01:08	-
Mean time for removing genome**	00:00:19	Not available	-
Time for consensus genome creation	00:00:47	Not available	-

First we compare the run-time and memory usage of pan-genome creation for the set of 43 *M. tuberculosis* genomes. PanCake requires pairwise genome comparisons by nucmer. Run-time and memory requirements for nucmer are listed separately as these can be run in parallel. We also evaluate the file size of the resulting pan-genome. We clock all available features (adding a genome, extracting part of a genome or the whole genome, remove a genome and constructing a consensus genome). * Extraction times for whole genomes and parts of sequences are equal. We extracted the interval 500-1000 for all genomes. ** Each of the 43 genomes was removed from the whole set

The memory requirements during the alignment construction are correlated with the elapsed time in most cases and are therefore lowest for seq-seq-pan, except for TBA. However, memory consumption of TBA will increase with the level of parallelization.

### Comparison with pan-genome tools

In addition to the set of reference genomes, PanCake requires pairwise alignments of all genomes to construct a pan-genome. In the case of our experiments with 43 *M. tuberculosis* genomes, the construction of ${\binom {43}{2}=903}$ pairwise alignments is required. For our comparison, we calculated these sequentially, but depending on the available hardware, this task can easily be parallelized. For this reason, we list the run-time and memory requirements of pairwise alignments with nucmer [24] separately (Table 6). Constructing the pan-genome with PanCake takes considerably longer than with seq-seq-pan. Also, the extraction of genomes or intervals of genomic sequences takes more time. The resulting pan-genome file from PanCake is smaller in size than the one created with seq-seq- pan. The reason for this difference in size and sequence extraction times is the strategy of PanCake of storing only the differences to a reference genome instead of the whole sequence for all genomes within a shared feature. Removing genomes and the generation of a consensus genome are features that are only provided by seq-seq-pan as listed in Table 1. PanCake detects and aligns sequence duplications within genomes and provides methods to compute core regions that are present in all aligned genomes. Arbitrary subsets of sequences can be extracted and singleton sequences that are only present in individual genomes can be identified [16]. Due to our choice to provide the results of seq-seq-pan in standard formats (XMFA, MAF) existing methods can be used for analysis and examination of alignment properties. For example, the maf_parse method of the Phast package [35] can be used to extract sub-alignments in specific regions or based on feature annotation files.

## Discussion

In this contribution, we introduced seq-seq-pan which enhances whole genome alignments by adding critical features for pan-genome data structures e.g. updating the set of genomes within the pan-genome. It provides a fast and simple construction process for whole genome alignments while optimizing the results for usage in subsequent analyses. The continuous merging of small unaligned blocks prevents the accumulation of sequences without context or position within the alignment and preserves the synteny of the original genomes, while the realignment of pairwise alignments avoids the introduction of additional repeats into the linear pan-genome representation. Both steps influence the composition of the linear consensus sequence and support its usage with mapping based methods such as read alignment.

The whole genome alignment format that we use as representation of a pan-genome in seq-seq-pan retains the full sequences and gaps for all aligned genomes in addition to meta-information about block borders. Therefore, it is not suitable to store the pan-genome efficiently. However, this format ensures loss-less and faster handling of the data. Further, it is thereby accessible by currently available downstream analysis tools without requiring subsequent novel tool implementations.

We demonstrate that the sort order of genomes does not substantially influence the result despite the sequential nature of our approach.

We compared seq-seq-pan with four whole genome aligners that offer alignment of non-collinear sequences. These tools use sophisticated methods for the identification of ortho- and even paralogs and conserved sequences. With these features, they identify similar but unrelated sequences within genomes, an aspect that is not considered in the field of pan-genomics. As we do not take such measures, we did not expect very high concordance between our results and the whole genome alignments. However, our comparison shows that our alignment differs as much from the results of progressiveMauve, progressiveCactus, Mugsy and TBA as their results differ among each other. Our approach is able to align a set of genomes much faster and with less memory usage than these whole genome alignment tools. Due to the focus on highly conserved sequences, some of these tools also provide a very fragmented alignment with many small blocks, which is prevented by the merge step in seq-seq-pan.

We compare our approach with currently available methods in terms of applicability and needed prerequisites (input data). For a detailed comparison, we chose PanCake as an approach by which a pan-genome can be constructed from a large set of genomes. We show that the construction of the pan-genome and using the structure for basic tasks requires substantially less time with seq-seq-pan than with PanCake. Some features, such as removing a genome from the pan-genome and the construction of a linear presentation of the pan-genome in the form of a consensus sequence, are not directly available in any other pan-genomics tool. For instance, the authors of PanCake focused on the analysis of core and accessory gene sets and therefore provide different functionalities.

In the time between November 30th, 2016 and January 20th, 2017 eight new *M. tuberculosis* genomes became available in the NCBI Ref-Seq database. This already highlights the importance of having the ability to extend a pan-genome structure. Methods such as the investigated whole genome alignment tools that constrain the user to start the alignment afresh with the increased number of genomes are at risk of reaching computational limits (some indications could be observed for Mugsy in the experiments already) which is mitigated by our iterative approach which quickly adds new sequences without having to rebuild previously calculated results. Furthermore, publicly available sets of genomes, such as the collection of “Complete Genomes” in the NCBI RefSeq database, are subject to change due to altered quality standards or the redefinition of reference genomes, such as the commonly used *M. tuberculosis* H37Rv strain. Therefore, it is essential that pan-genome representations also provide the feature to easily remove genomes from the initial set without impacting the remaining genomes. Most of the evaluated tools do not provide methods for updating a constructed pan-genome. Particularly research like molecular surveillance, where new data is continuously analyzed and incorporated, depends on data structures that allow the integration of an up-to-date set of genomes.

## Conclusions

In summary, we present a data structure for the representation of pan-genomes that provides a unique set of features needed for efficiently working with collections of related sequences and that can be integrated with existing methods for visualization and subsequent analyses.

## Additional files

Additional file 1 Supplementary Information for “seq-seq-pan”: Building a computational pan-genome data structure on whole genome alignment”. Additional file 1 provides details on implementation of the sequential workflow for whole genome alignment. (PDF 183 kb)

Additional file 2 Supplementary Tables for “seq-seq-pan: Building a computational pan-genome data structure on whole genome alignment”. Additional file 2 provides detailed description of the used datasets, including accession numbers and sort order. (XLSX 83 kb)

## Abbreviations

LCB: Locally collinear block
NGS: Next generation sequencing
WGA: Whole genome alignment
