# Supplementary material for: seq-seq-pan: building a computational pan-genome data structure on whole genome alignment
Source: BMC Genomics. 2018 Jan 15;19:47. doi: 10.1186/s12864-017-4401-3 (PMC5769345; doi:10.1186/s12864-017-4401-3)
Supplement: Supplementary file 1 — Supplementary Information for “seq-seq-pan”: Building a computational pan-genome data structure on whole genome alignment”. Additional file 1 provides details on implementation of the sequential workflow for whole genome alignment. (PDF 183 kb) [file 12864_2017_4401_MOESM1_ESM.pdf]

# Supplement for seq-seq-pan: Building a computational pan-genome data structure on whole genome alignment

Christine Jandrasits[1], Piotr W Dabrowski[1], Stephan Fuchs[2] and Bernhard Y Renard[1*]

## Implementation details

This document contains implementation details for seq-seq-pan. The order of all steps for each iteration of the sequential workflow is depicted in Fig. S1. For the alignment of pairs of genomes we use progressiveMauve (snapshot 2015-02-13). All other parts of the sequential workflow are implemented in Python3.4. For performance reasons, the consensus construction step was additionally implemented in Java8. We use the following Biopython (version 1.68) modules ([1]) in our workflow: SeqIO, Seq and SeqRecord, for reading, writing and manipulation of single sequences and pairwise2 for aligning two sequences in the Realignment step. For performance reasons we use blat ([2]) for the alignment of large sequences in the Realignment step.

For a straightforward construction of a pan-genome from a set of genomes, we combined all steps with the workflow management software Snakemake ([3]). The pipeline can be parametrized to include the optional merging steps and all necessary steps are determined automatically in each iteration. The output is composed of the final alignment of all input genomes and the corresponding consensus genome including index files necessary for following analyses and further updates of the pan-genome.

[*]Correspondence: RenardB@rki.de

**Author details**

[1]Robert Koch Institute, Nordufer 20, 13353, Berlin, Germany. [2]Robert Koch Institute, Wernigerode Branch, Burgstrae 37, 38855, Wernigerode, Germany.

**References**

1. Cock, P.J., Antao, T., Chang, J.T., Chapman, B.A., Cox, C.J., Dalke, A., Friedberg, I., Hamelryck, T., Kauff, F., Wilczynski, B., De Hoon, M.J.L.: Biopython: freely available python tools for computational molecular biology and bioinformatics. Bioinformatics **25**(11), 1422–1423 (2009)
2. Kent, W.J.: Blatthe blast-like alignment tool. Genome research **12**(4), 656–664 (2002)
3. Köster, J., Rahmann, S.: Snakemake - a scalable bioinformatics workflow engine. Bioinformatics **28**(19), 2520–2522 (2012)

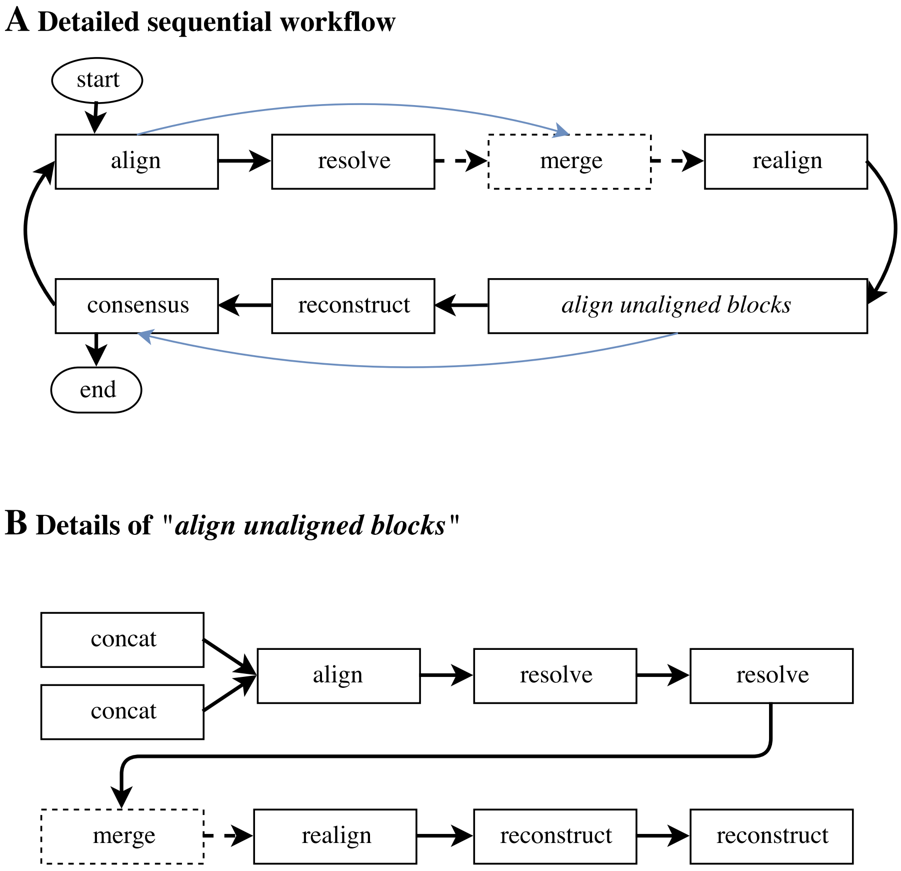


**Figure S1 Detailed sequential workflow.** Blocks represent steps in the workflow, dashed ones are optional. The first iteration is different and outlined with the blue lines. Subsequent iterations are represented with black arrows.
**(A)** After aligning two genomes - two original ones in the first iteration and a consensus genome with another original one in all following - the result is optimized with an optional merge step and local realignment of sequences around consecutive gap stretches. Initially unaligned single-sequence blocks are aligned again and all resulting blocks are joined with the blocks resulting from the original alignment. Then the consensus genome is constructed using the optimized alignment. The consensus genome is aligned with the next original genome. Alignments over block borders in the consensus sequence are resolved and the alignment is optimized by merging and realignment. Again, initially unaligned blocks are aligned separately. After joining of all LCBs the full alignment of all genomes is reconstructed.
**(B)** For aligning initially unaligned sequences of each genome, the same methods as for the main alignment are used. All unaligned sequence parts of each genome are sorted and concatenated with 'N' stretches as delimiters. The alignment of these two constructed sequences again results in several LCBs. Alignments stretching over borders of concatenated blocks are resolved successively for each of the genomes. The alignments are optionally optimized with the merging and realignment steps. After that the alignment of the original sequences of both genomes is sequentially reconstructed.
